# Development of *Ophiocordyceps sinensis* through Plant-Mediated Interkingdom Host Colonization

**DOI:** 10.3390/ijms160817482

**Published:** 2015-07-30

**Authors:** Wei Lei, Guren Zhang, Qingyun Peng, Xin Liu

**Affiliations:** 1 Laboratory of Cardiovascular Diseases, Guangdong Medical College, Zhanjiang 524001, China; E-Mail: leiwei2006@126.com or shuixiaor@gmail.com; 2 Food and Health Engineering Research Center of State Education Ministry, School of Life Sciences, Sun Yat-sen University, Guangzhou 510275, China; E-Mails: zhanggr@mail.sysu.edu.cn (G.Z.); p287931475@126.com (Q.P.)

**Keywords:** *Ophiocordyceps sinensis*, interkingdom colonization, plant, caterpillar, development

## Abstract

*Ophiocordyceps sinensis* is a well-known entomogenous and medicinal fungus. After its anamorphs parasitize the larvae of the genus *Thitarodes*, fruit-bodies may form to be used as medicine. However, its developmental mechanisms remain unknown. The distribution of *O. sinensis* was determined in different tissues of the *Thitarodes* larvae and the dominant plant species using real-time quantitative polymerase chain reaction (qPCR) and fluorescence *in situ* hybridization (FISH) technique, respectively. We found that more fungal material was located in plants than in larvae, especially in *Ranunculus tanguticus*. A considerable amount was detected in larval intestinal-wall and plant roots. It is suggested that plants are the potential hosts of *O. sinensis*, which modifies our understanding of the life cycle of *O. sinensis* and indicates that the phytophagous larvae may become infected as they feed. Our research may contribute to the study of systematic evolution and population ecology of *O. sinensis*, elucidate its developmental mechanism and promote sustainable harvesting.

## 1. Introduction

*Ophiocordyceps sinensis* (Syn. *Cordyceps sinensis*) is a well-known entomogenous and medicinal fungus, and it is naturally distributed in mountain shrubbery and meadows on the Tibetan Plateau [1,2]. The fungal fruit-body with sclerotium has been widely used to provide positive support for the lungs, the kidneys and the immune system [3]. Due to its increasing popularity in the Asian market and complete dependence on natural yield, refined *O. sinensis* products now cost as much as US $ 60,000 per kilogram. The life cycle of this fungus includes asexual and sexual phases [4]. After its anamorph *Hirsutella sinensis* develops in the larvae of the genus *Thitarodes* (caterpillars) which live on the plant roots in the underground tunnels, the fruit-bodies (stromata) may form under appropriate conditions [5,6,7]. However, its developmental mechanisms are almost completely unknown, such as infection pathway and pathogenic status. The *Thitarodes* insects are generally considered to be the only hosts of the *O. sinensis* during its asexual cycle [8]. In view of certain previous reports regarding interkingdom host jumping of the genus *Cordyceps* based on the phylogenetic analyses [9], we decided to study facultative parasitism in *O. sinensis*, which may contribute to reveal the occurrence mechanism of this precious fungus. Nevertheless, its mycelia grow only very slowly on agar media under certain conditions [10,11], so it has not been available directly from the host and environment so far. Thus, *in situ* investigation coupled with molecular detection becomes a promising approach.

Endophytic and parasitic fungi show a certain degree of host specificity, colonizing and infecting a class of organism, a particular species, or even a particular tissue. Host specificity may be the natural outcome of co-evolutionary conservation between hosts and parasites. However, in order to survive and disperse their propagules, these fungi also need frequent encounters and to infect new host individuals. Two hypotheses have been put forward to interpret the phenomenon of host shifts [12,13]. These are the host relatedness hypothesis and the host habitat hypothesis. According to the former, phylogenetically related hosts harbour the same or similar fungi. It can explain why hosts that are related at the lower taxonomic levels possess similar parasites [13,14]. It is universally found that some related fungi infect a group of related hosts. The other hypothesis concerns hosts at higher taxonomic levels; host shifts depend on microhabitat and suitability of food. This hypothesis provides a reasonable explanation for the phenomenon in which a fungus or a group of related fungi can colonize and utilize distantly related organisms [15,16]. These two hypotheses, which do not contradict each other, are applicable to different situations. Numerous reports have addressed ecological overlaps in fungus-host interactions [17,18].

In the present study, the distribution of *O. sinensis* was detected both quantitatively and visually in different tissues of caterpillars and dominant plants by real-time quantitative polymerase chain reaction (qPCR) and fluorescence *in situ* hybridization (FISH) technique, respectively. Thus, we propose an inter-kingdom colonization by *O. sinensis* of a caterpillar and a plant and discuss the developmental mechanism of the fungus in the plant.

## 2. Results

### 2.1. Quantificational Detection of O. sinensis Using Quantitative Polymerase Chain Reaction (qPCR)

**Figure 1:**
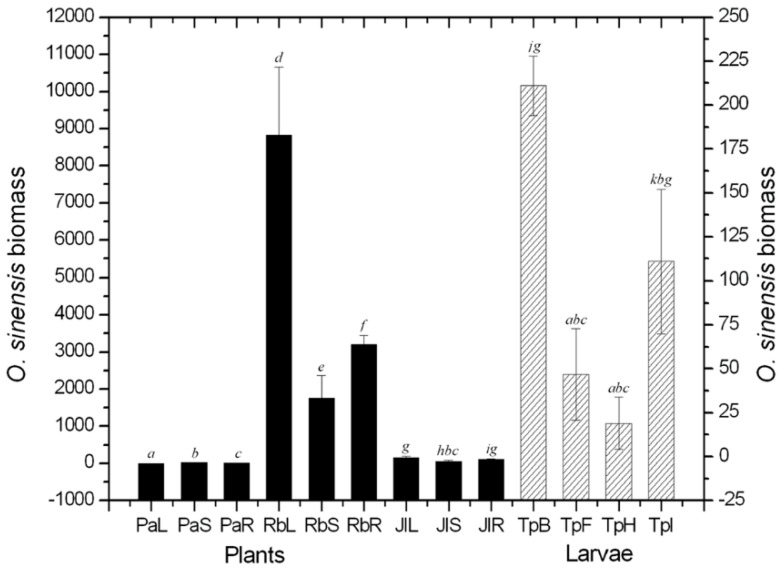
Biomass of *O. sinensis* in different tissues of the caterpillars and the dominant plants in Nyingch prefecture. PaL: *Primula alpicola* leaf; PaS: *P. alpicola* stem; PaR: *P. alpicola* root; RbL: *Ranunculus tanguticus* leaf; RbS: *R. tanguticus* stem; RbR: *R. tanguticus* root; JlL: *Juncus leucanthus* leaf; JlS: *J. leucanthus* stem; JlR: *J. leucanthus* root; TpB: *Thitarodes pui* body-wall; TpF: *T. pui* fat-body; TpH: *T. pui* haemolymph; TpI: *T. pui* intestinal-wall. Differences between means at *p* < 0.05 are indicated by different letters (bars with the same letter are not significantly different).

**Figure 2:**
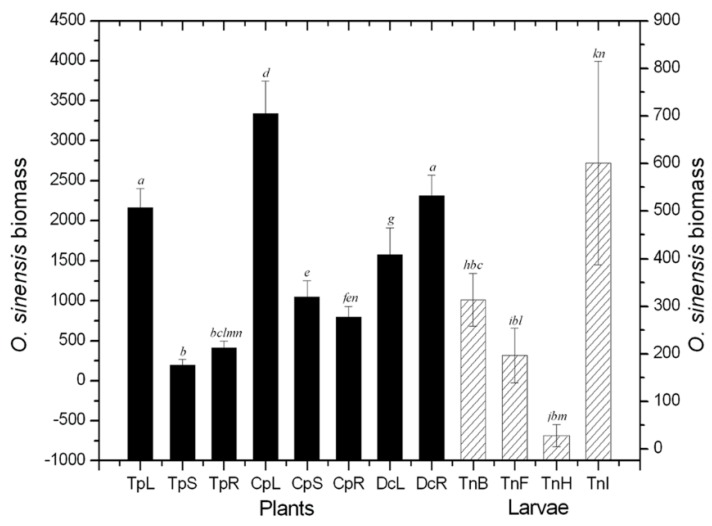
Biomass of *O. sinensis* in different tissues of the caterpillars and the dominant plants in Damxung prefecture. TpL: *Trollius pumilus* leaf; TpS: *T. pumilus* stem; TpR: *T. pumilus* root; CpL: *Chamaesium paradoxum* leaf; CpS: *C. paradoxum* stem; CpR: *C. paradoxum* root; DcL: *Deschampsia caespitosa* leaf; DcR: *D. caespitosa* root; TnB: *Thitarodes* *namensis* body-wall; TnF: *T.* *namensis* fat-body; TnH: *T.* *namensis* haemolymph; TnI: *T.* *namensis* intestinal-wall. Differences between means at *p* < 0.05 are indicated by different letters (bars with the same letter are not significantly different).

The ability of qPCR to quantify fungal biomass was validated by pure cultures of *O. sinensis*, and the determination coefficient was found to be *R*^2^ = 0.999519. After the quantitative assay, a certain amount of *O. sinensis* was detected in all caterpillar and plant tissues, although the values for the *Primula alpicola* leaves, stems and roots were so low as to be almost negligible in Figure 1. After investigating in Nyingch (southern Tibetan Plateau, 4156 m altitude, 29°36′N, 94°35′E) and Damxung (northern Tibetan Plateau, 4858 m altitude, 30°36′N, 91°06′E) (Figure 1 and Figure 2), the colonization content of *O. sinensis* in plants was probably larger on average than that observed in the caterpillars by the same volume of tissues. Moreover, a larger amount of *O. sinensis* were found in the intestinal-walls, from where it could enter the coeloms of the caterpillars as they feed plants.

The amount of *O. sinensis* from plants in southern Tibet was much higher than in northern Tibet, indicating its colonization level in plants may be positively related to temperature. Colonization reached its highest value in the leaves of *Ranunculus tanguticus*, implying that plants, especially *R. tanguticus*, might be a significant host of *O. sinensis* (Figure 1). The total amount of *O. sinensis* in caterpillars in the southern region was lower than that in north, where the fungal material in the larval body-walls was significantly lower than in the intestinal-walls.

### 2.2. In Situ Investigation of O. sinensis Using Hybridization (FISH)

**Figure 3:**
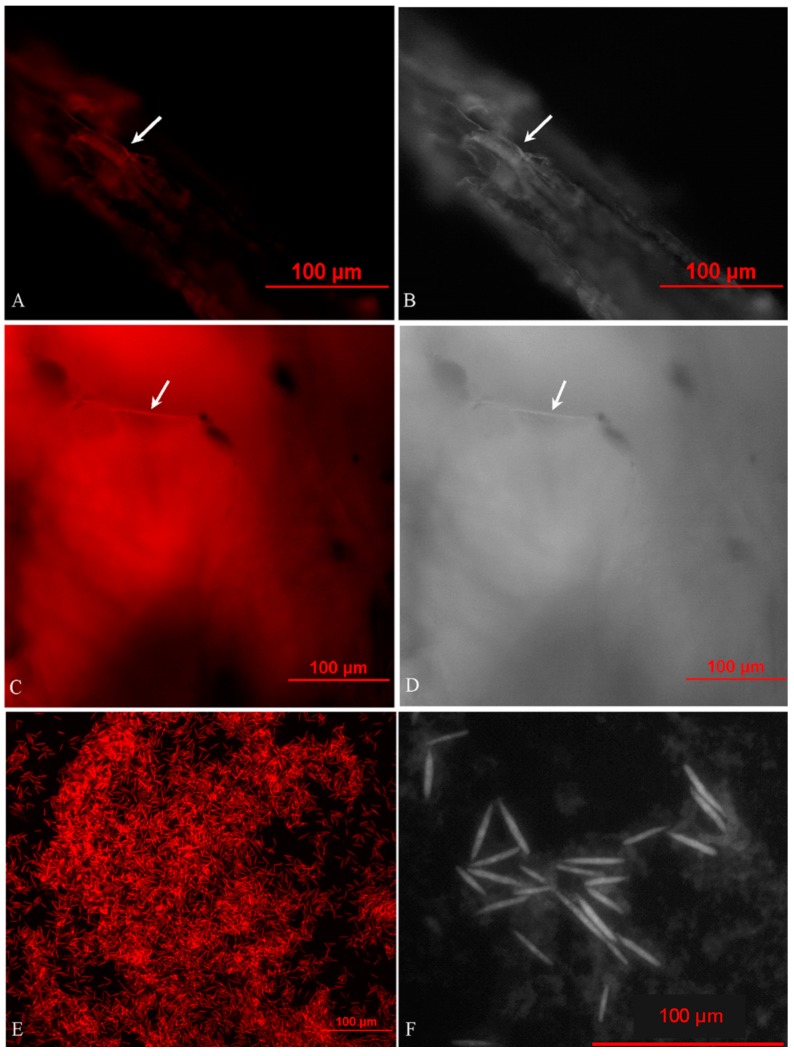
Fluorescence *in situ* hybridization (FISH) with the specific *O. sinensis* probe (OSP) of *O. sinensis*. (**A**) Cy5-OSP-labeled micrograph of the *O. sinensis* hyphae in the plant root; (**B**) light micrograph of the *O. sinensis* hyphae in the plant root; (**C**) Cy5-OSP-labeled micrograph of the *O. sinensis* hyphae in the caterpillar intestinal-wall; (**D**) light micrograph of the *O. sinensis* hyphae in the caterpillar intestinal-wall; (**E**) Cy5-OSP-labeled micrograph of the *O. sinensis* endosymbionts in the caterpillar haemolymph; (**F**) light micrograph of the *O. sinensis* endosymbionts in the caterpillar haemolymph. The red hybridization signal is indicated with an arrowhead.

*O. sinensis* filaments and patchy areas were observed in the caterpillar and plant tissues with fluorescence and light microscopies, and they denoted either the hyphae or their agglomeration (Figure 3A–D). *O. sinensis* was specifically recognized in the caterpillars during initial development. Yeast-like endosymbionts of the same identity coexisted with the hyphae (Figure 3E). The particles of the endosymbiont were about 30~50 μm long (Figure 3F). *O. sinensis* was detected throughout the bodies of the caterpillars and plants. This is consistent with the results of qPCR. Some hyphae were embedded in tissues of the hosts, indicating that these hyphae might be endophytic in caterpillars and plants. *O. sinensis* was confirmed as having colonized the plants and insects in such a manner that the fungus would survive and multiply.

### 2.3. Effect of Culture with Plant Extracts on O. sinensis Mycelia

All plant extracts promoted the growth of *O. sinensis* colonies. This suggests that *O. sinensis* can take in the plant nutrition and survive in the tissues of plants, at least the tissues of *R. tanguticus*, *Juncus leucanthus*, and *P. alpicola*. The difference between above-ground tissues extracts (stems and leaves) and the root extracts varied by species. Colony size was largest on the media including *R. tanguticus* extracts. This result was consistent with those of the molecular assays. However, the colony morphologies and sizes on the plant extract media were smaller than those on the PDA media, indicating that high sugar content was likely propitious to the growth of *O. sinensis* mycelia.

## 3. Discussion

*O. sinensis* is detected qualitatively by endpoint PCR techniques including conventional PCR, touch-down PCR and nested PCR [19,20], whose precision, sensitivity and stability all need to be further improved, especially for detection of complex samples. qPCR can measure the copy number of target DNA fragments with the help of intercalating dyes, such as Synergy Brands (SYBR) green [21]. Use of qPCR-based methods is common in microecology since 2000, and mechanisms are analyzed quantitatively and accurately [22,23]. In this study, the amount of *O. sinensis* was quantified successfully in different tissues of caterpillars and dominant plants from the main production areas Nyingch and Damxung. These two prefectures are representative locations for *O. sinensis* in the southern and northern plateau respectively. The fungus was found to a greater extent in plants than in caterpillars, which had obvious regional and narrowly distributed characteristics [4,24,25]. A considerable amount of *O. sinensis* was found in plants leaves, implying that the fungus can spread with the wind even farther away than by host insects and this may help to interpret why *O. sinensis* distributes wildly from north to south on the Tibet Plateau. Cultivation in media containing plant extracts showed that plant tissues could provide sufficient nutrients for the survival of *O. sinensis*, especially the root extracts of *R. tanguticus*. However, the highest amount of *O. sinensis* was found in the leaves of *R. tanguticus* by qPCR detection. This difference can be explained as follow: in nature the roots grow in the soil, where humic acid, heavy metal ions, and other chemical materials and pathogenic microorganisms can restrain the growth of *O. sinensis* [26]; in artificial media, obviously, such inhibitors are absent and, consequently, the fungus grows better. In addition, in the southern Tibet Nyingch with the abundant vegetation, the relative amount of *O. sinensis* in larval body-walls was higher than in those from the northern region Damxung, and meanwhile it was relatively lower in the intestine walls. Other studies [27] have shown that in the northern plateau much less fungal material is present in the soil than in the southern region. It can be concluded that *O. sinensis* is greatly affected in the larval cuticle by the soil. The amount in the intestine comes from the associated plants.

Under *in situ* investigation using FISH, *O. sinensis* hyphae were observed in all tissues of caterpillars and plants, coupled with yeast-like endosymbionts in the haemolymph, all which revealed the endoparasitic morphology of *O. sinensis* in its hosts [28]. This is likely because hyphae are generally the best means to adhere the solid by which the organism can take in nutrition and reproduce asexually; besides, the yeast-like endosymbionts, derived from the filamentous lineage due to losing some genes essential for hyphal growth, appear to adapt to the intracellular habit of insects and function in sterol utilization and nitrogen recycling for the hosts [29]. Although other fungi, for example *Beauveria bassiana*, are capable of infecting insects during its endophytic growth in plants, the ability of host-jumping is of primary importance for the highly prized medicinal fungus *O. sinensis*, and furthermore this fungus is a psychrophilic one that requires strict culture condition and grows quite slowly on agar media. Because of the dense wild vegetation in its natural habitat, germplasm resources of this fungus are nearly infinite. Thus, the population of the host caterpillar should be a limiting factor for wildlife conservation and sustainable harvest of *O. sinensis*, in contrast with previous reports based generally on experience [30,31,32]. A new strategy should be established to promote a reasonable utilization of this fungus by increasing the density of these caterpillars and suitable plants. Some effective measures have already been enacted, and others are in progress now. They include optimizing the oviposition and feeding habits of the host caterpillar in cultivated *Thitarodes* insects.

All of these results have consistently implied that it is highly necessary to add a step covering host jumping through plants to our understanding of the life cycle of *O. sinensis*. This is an important complementary modification for our understanding of the developmental process of *O. sinensis*. In accordance with the origins and evolution of entomopathogenicity in fungi, *O. sinensis* seems to be able to jump from plants to insects and back onto a fungal host [33]. The strong correlation between caterpillars and the local plants in a suitable soil microhabitat is likely to promote host switching [34]. The results of this study support the host habitat hypothesis, as did those of the phylogenetic analysis of the genus *Cordyceps* [35].

**Figure 4:**
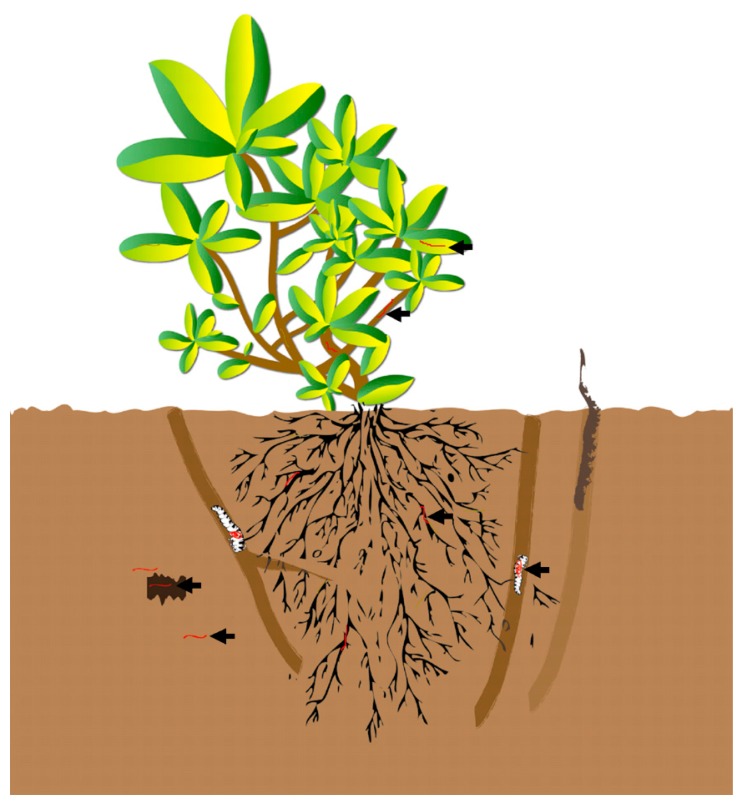
Relationships of *O. sinensis* with plants and caterpillars. *O. sinensis* are indicated with the red line and denoted by the arrows.

In addition, because *O. sinensis* can colonize plants, which are the caterpillars’ food, it may be that the host caterpillars become infected as they eat. The infected caterpillars then, under appropriate conditions, become muscardine cadavers and transform into *O. sinensis* teleomorphs with the valuable fruit bodies. To sum up, a novel insight concerning interkingdom colonization of insects and plants by *O. sinensis* is here presented (Figure 4). This may contribute to the study of the systematic evolution and population ecology of this organism and may lay a crucial foundation for the improved understanding of the mechanism by which *O. sinensis* develops.

## 4. Experimental Section

### 4.1. Materials

*O. sinensis* mycelia were isolated from the sclerotia of natural *O. sinensis* specimens gathered from Nyingch (southern Tibet, 4156 m altitude, 29°36′N, 94°35′E) and Damxung (northern Tibet, 4858 m altitude, 30°36′N, 91°06′E), and then cultured at 18 °C. In the Nyingch prefecture, the larvae of *Thitarodes pui*, and the main plants in the *O. sinensis* habitat including *R. tanguticus*, *J. leucanthus*, *P. alpicola*, were collected [20]. Another group of samples was obtained from Damxung prefecture, such as *T. namensis* larvae, and the three plants *Trollius pumilus*, *Chamaesium paradoxum*, *Deschampsia caespitosa*. Most of the experiments in the current study were carried out in “Characteristic Resources Scientific Workstation of Sun Yat-sen University”, which is located in the habitat in order to ensure accuracy and dependability of the methods and results.

### 4.2. DNA Extraction and qPCR Assay

Genomic DNA was extracted from pure cultures of *O. sinensis*, from four types of caterpillar tissues, including body-wall, fat-body, hemolymph, and intestinal-wall, and from the roots, stems, and leaves of the dominant plants (*R. tanguticus*, *J. leucanthus*, *P. alpicola*, *T. pumilus*, *C. paradoxum*, and *D. caespitosa*) using the AxyPrep Multisource Genomic DNA Miniprep Kit (Axygen Scientific Inc., Union City, CA, USA). First of all, these materials were washed with 75% ethanol solution for 1 min, so as to eliminate the negative impact of the *O. sinensis* population on the surfaces of the insects and plants.

qPCR quantitative detection of *O. sinensis*, based on the internal transcribed spacer rDNA (nrITS), was carried out according to our developed method [36]. nrITS levels were quantified by qPCR reaction using SYBR Green PCR Master Mix (2×) (Toyobo, Osaka, Japan) with the species-specific primer pair (F: 5′-GCAGTGGCATCTCTCAGTCA-3′; R: 5′-GCATTTCGCTGCGTTCTT-3′). Samples were amplified with the Applied Biosystems 7500 Real-Time PCR System (Life Technologies, Foster, CA, USA).

The efficiencies and accuracy of the qPCR assays were estimated from standard calibration curves based on serial 5-fold dilutions of plasmid standards with the 390.5 ng·μL^−1^ of DNA initiative sample. The absolute quantification of the target nrITS region was performed based on the calibration curves using the above method. The resulting concentration was converted into copy numbers of the template DNA, which was used for quantification of *O. sinensis* biomass [36,37]. Copy numbers were calculated for the amplified fragments according to the formula:

Copy number = (DNA amount × 6.022 × 1023)/(length × 1 × 109 × 660)
(1)

### 4.3. Hybridization (FISH) Protocol for in Situ Detection of O. sinensis

FISH investigation of *O. sinensis* was performed as described previously [38,39], the *O. sinensis*-specific oligonucleotide probe (OSP: 5′-CAGGCACGTCAGCGCTCG-3′) was synthesized commercially by Life Technologies Inc. (Guangzhou, China), and the 5′ end labeled with Cy5 reactive dye (fluorescence excitation wavelength 675 nm, red light).

The materials were sampled from the four caterpillar tissues (body-wall, fat-body, hemolymph, and intestinal-wall), and from the roots, stems, and leaves of the dominant plants *T. pumilus*, *C. paradoxum*, *D. caespitosa*, *R. tanguticus*, *J. leucanthus*, and *P. alpicola*. After being washed three times with autoclaved distilled water, these samples were sectioned and fixed in Carnoy’s fluid for 2 h. They were then washed twice with phosphate-buffered saline (PBS) and re-suspended in the solution with equal volumes of PBS and absolute ethanol. Air-dried samples were treated with the lywallzyme solution (Guangdong Institute of Microbiology, Guangzhou, China) at a final concentration of 10 mg·mL^−1^ and incubated for 10 min at 30 °C. After dehydration in 50%, 80%, and 96% ethanol for 3 min, hybridizations were conducted in 20 μL hybridization buffer (5× SSC, 1× Denhardt’s solution, 100 μg·mL^−1^ milt DNA, 10% dextran sulfate, deionized formamide, and 5 ng OSP probe) at 42 °C for 16 h using a programmable temperature controlled system (StatSpin^®^ ThermoBrite, Abbott, Norwood, MA, USA). Then, the samples were washed for 10 min at 42 °C in pre-warmed 1× SSC buffer. They were then counterstained using the nucleic acid-specific dye DAPI for 20 min at a concentration of 0.05 μg·mL^−1^. The prepared samples were observed to screen the positive ones under Eclipse 80i epifluorescence microscopy (Nikon, Tokyo, Japan) with 4,6-diamidino-2-phenylindole (DAPI) and Cy5 filters connected to a Nikon digital camera (DS-Qi1Mc-U2, Nikon, Tokyo, Japan), and the images were orderly captured under blue (λ 477 nm), red (λ 675 nm) and full-wavelength emission light, respectively. After comparing with the blue and red components of the same sample in order to exclude the false positive, these images were merged and analyzed using NIS-Elements BR 3.1 software (Nikon, Tokyo, Japan) [38].

### 4.4. Effect of Plant Extracts on O. sinensis Mycelia

Ten grams of plant materials was prepared from leaves, stems, and roots of *R. tanguticus*, *J. leucanthus*, and *P. alpicola*. In order to eliminate false positives from *O. sinensis* living on the surfaces of the plants, these materials were washed with 75% ethanol solution for 1 min. After being immersed in 200 mL of double-distilled water for 4 h, the plant materials were extracted over a slow fire (75 °C) for 30 min. This was followed by filtration, and 100 mL was added to each of the filter residues. Samples were then decocted for 20 min to produce more filtrate. The two filtrates were combined and concentrated to 10.0 g·mL^−1^. In this way, six plant water extracts were sterilized and made into six groups of culture media (2.0 g·mL^−1^), with five plates in each group. Equivalent amounts of *O. sinensis* mycelia were inoculated onto these plant media with PDA media as positive control and pure agar media as negative control. This was followed by culture at 18 °C for three months.

### 4.5. Data Analyses

SPSS software was used for statistical analyses. In order to determine the relationship between fungal nrITS copy numbers and *C*_t_ values, liner regression and coefficients of determination (*R*^2^) value were calculated using the Proc Reg method. To evaluate the effects of fungi on the different colonization outcomes of *O. sinensis* in the different tissues of host, the levels of significance were analyzed using Least Significant Difference (LSD)-*t* test in pairwise comparisons and different letters indicated a significant difference at *p* < 0.05.
